# Lesion conspicuity assessment of prone 40-keV virtual monoenergetic imaging in arterial-phase photon-counting CT for breast cancer: a retrospective comparison with DCE-MRI

**DOI:** 10.1007/s11604-026-01979-7

**Published:** 2026-03-31

**Authors:** Yusuke Kawasaki, Maika Nakajima, Kota Yokoyama, Emi Yamaga, Makoto Kato, Leona Katsuta, Ken Yamagiwa, Hiroto Hada, Takumi Hiraishi, Hirofumi Yamada, Junichi Tsuchiya, Susumu Kirimura, Iichiroh Onishi, Tomoyuki Aruga, Ukihide Tateishi

**Affiliations:** 1 Department of Diagnostic Radiology and Nuclear Medicine, Institute of Science Tokyo, 1-5-45, Yushima, Bunkyo-ku, Tokyo, 113-8510 Japan; 2 Radiology Center, Institute of Science Tokyo Hospital, Bunkyo-ku, Tokyo, Japan; 3 Department of Breast Surgery, Institute of Science Tokyo, Tokyo, Japan; 4 Department of Comprehensive Pathology, Graduate School of Medical and Dental Sciences, Institute of Science Tokyo, Tokyo, Japan

**Keywords:** Breast neoplasms, Breast imaging, Computed tomography, Photon-counting, Magnetic resonance imaging, Virtual monoenergetic imaging

## Abstract

**Purpose:**

This study investigated whether contrast enhanced whole-body photon-counting detector (PCD)-CT performed for staging in the prone position can provide breast lesion conspicuity comparable to dynamic contrast-enhanced T1-weighted imaging (DCE-T1WI).

**Methods:**

This single-center, retrospective diagnostic feasibility study included 22 women with surgically confirmed primary breast cancer who underwent preoperative prone PCD-CT and DCE-MRI. Then, 40- and 70-keV virtual monoenergetic image (VMI) from a single arterial-phase PCD-CT acquisition were reconstructed. Three readers independently evaluated 40- and 70-keV VMI and DCE-T1WI. The primary endpoint was lesion conspicuity. The secondary endpoints included, size error, detection rate, background parenchymal enhancement (BPE), subjective noise, and reading time.

**Results:**

Lesion conspicuity was highest on 40-keV VMI, which outperformed 70-keV VMI and DCE-T1WI. Absolute size error was numerically larger for 40-keV VMI than for DCE-T1WI, but this difference was not statistically significant. Directional error indicated that 40-keV VMI significantly underestimated lesion size relative to DCE-T1WI. Detection rates showed an overall difference among modalities, but pairwise differences were not statistically significant. Further, 40-keV VMI had a higher BPE and a greater perceived noise than 70-keV VMI, and it required a longer reading time than DCE-T1WI. The radiation dose did not differ between VMI energy levels as both were reconstructed from the same acquisition.

**Conclusion:**

Arterial-phase 40-keV VMIs from prone PCD-CT demonstrated the highest lesion conspicuity and showed better detection capability than 70-keV VMI. In addition, their performance was comparable to or superior to DCE-T1WI. This finding was achieved despite trade-offs in increased noise, BPE and size underestimation. Thus, 40-keV VMI may serve as a useful complementary modality for local breast assessment and may be considered a potential alternative for lesion localization when MRI is not feasible, despite potential underestimation of lesion extent.

**Supplementary Information:**

The online version contains supplementary material available at 10.1007/s11604-026-01979-7.

## Introduction

Breast cancer is among the most common malignancies and a major cause of cancer-related mortality among women [1]. An accurate preoperative local assessment plays a key role in determining surgical planning and therapeutic strategies [2]. At present, owing to its high sensitivity for lesion detection, mapping of disease extent, and identification of multifocal or multicentric disease, dynamic contrast-enhanced magnetic resonance imaging (DCE-MRI) is considered as the gold standard for local disease evaluation [3, 4].

Conventional CT has been considered suboptimal for soft-tissue contrast in breast imaging. In contrast, spectral CT—including dual-energy CT (DECT)—has gained attention as a promising complementary modality. Low-energy virtual monoenergetic images (VMIs), particularly those around 40 keV, enhance iodine attenuation and improve contrast-to-noise ratio (CNR) and lesion conspicuity across a range of diseases, including breast cancer [5–7], although this benefit is accompanied by increased image noise. Photon-counting detector (PCD)-CT is a technological advancement that offers fine spatial resolution and true energy discrimination [8–10]. This enables the generation of multiple VMIs (e.g., 40- and 70-keV) from a single arterial-phase acquisition without increasing radiation exposure [11, 12]. In addition, the photon-counting architecture reduces electronic noise and improves low-energy signal fidelity, which helps preserve image quality even at low-keV reconstructions [13, 14].

Beyond improved soft-tissue contrast, PCD-CT can mitigate several practical and logistical limitations that are associated with MRI. It enables simultaneous whole-body staging and local breast assessment in a single session [7, 15, 16], provides quantitative spectral parameters, such as iodine density and virtual non-contrast images for objective analysis [12, 17], and achieves a high spatial resolution suitable for identifying fine structures including microcalcifications—an area where MRI is less optimal [15, 18]. Accordingly, PCD-CT may be a rapid and accessible alternative when MRI is delayed, contraindicated, or limited in terms of availability [16].

Prior studies have suggested that low-keV VMI from spectral CT improves the conspicuity of enhancing breast tumors by increasing iodine attenuation at low energy levels [6]. In cohorts directly compared with MRI, low-keV VMI has also been reported to improve the estimation of lesion extent relative to conventional 120-kVp CT, including for intraductal components [7]. These prior reports indicate that low-keV VMI may be useful for CT-based preoperative assessment in selected settings, while preserving the practical advantage of combining local assessment and systemic staging in a single examination. However, only a few studies have directly compared contrast-enhanced breast PCD-CT protocols, including VMI-based reconstructions with clinical DCE-MRI [7, 19]. Whether the lesion conspicuity of arterial-phase PCD-CT is comparable to that of MRI remains insufficiently investigated.

The primary aim of this study was to compare 40-keV PCD-CT VMIs with dynamic contrast-enhanced T1-weighted imaging (DCE-T1WI) in participants with surgically confirmed primary breast cancer. A secondary aim was to examine potential performance differences between 40 and 70-keV VMIs.

To address these aims, cross-modality comparisons were performed among 40-keV VMI, 70-keV VMI, and DCE-MRI with respect to lesion conspicuity, size error, detection rate, background parenchymal enhancement (BPE), subjective noise, and reading time. Based on prior breast spectral CT studies, 40-keV was selected as the primary low-energy setting to maximize lesion conspicuity [6], while 70-keV was included as a reference level approximating conventional 120 kVp CT image appearance [20]. Importantly, the evaluated PCD-CT datasets were obtained as part of routine whole-body contrast-enhanced CT scan performed for metastatic staging—a study that is already universally incorporated into the preoperative workup of breast cancer. The ability to extract diagnostically useful local breast information from this same acquisition, without requiring a separate dedicated breast MRI, is a central motivation for this investigation.

## Methods

### Study design and participant selection

The institutional review board approved this single-center retrospective study (approval no.: I2024-084), and the requirement for a written informed consent was waived.

At our institution, PCD-CT in the prone position has been selectively performed in clinical practice when detailed local breast assessment, such as evaluation of microcalcifications or tumor extent, was specifically requested by the referring clinicians. Accordingly, patients with histopathologically confirmed primary breast cancer who underwent preoperative staging with contrast-enhanced PCD-CT between October 2024 and May 2025 were retrospectively examined as a real-world clinical cohort.

The inclusion criteria were as follows: (a) availability of both preoperative contrast-enhanced PCD-CT and breast MRI, (b) presence of an arterial-phase series for PCD-CT, and (c) both examinations performed in the prone position. The only exclusion criterion was a history of neoadjuvant chemotherapy (NAC) before PCD-CT.

### PCD-CT acquisition

All CT scan examinations were performed using a photon-counting detector CT scanner (NAEOTOM Alpha, Siemens Healthineers, Erlangen, Germany). Images were reconstructed using Quantum Iterative Reconstruction (QIR level 2) with kernel Qr40, a slice thickness of 1.0 mm, a reconstruction increment of 1.0 mm, and a matrix size of 512 × 512. Based on previous DECT scan studies showing superior lesion contrast and conspicuity at this energy, 40-keV VMI was selected as the primary low-energy setting [6]. Spectral reconstructions were used to generate VMIs at 40- and 70-keV. All spectral reconstructions were performed on *syngo*.via (Siemens Healthineers, Erlangen, Germany).

**Fig. 1:**
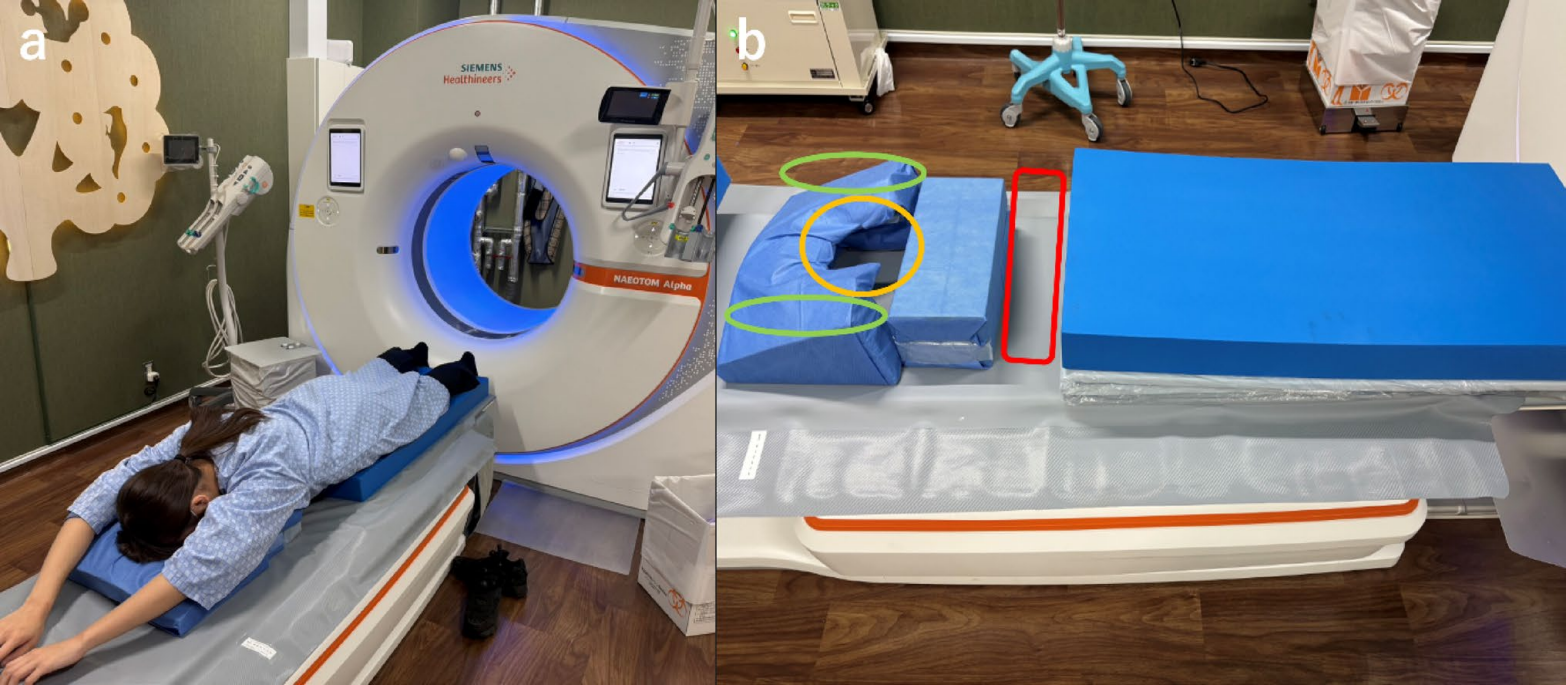
Prone positioning protocol for CT acquisition. **a** Patient positioned prone on the CT table using the standardized support setup. **b** Support arrangement and intended positioning. The face is placed within the head support (yellow circle), the arms are positioned on the arm supports (green circles), and the breasts are allowed to hang freely in a dependent, non-compressive configuration in the designated opening (red rectangle) to minimize deformation

At our institution, prone PCD-CT was performed using a standardized support setup designed to minimize breast deformation. Both breasts were positioned in a dependent, non-compressive configuration (Fig. 1).

### Contrast protocol

The examinations were conducted based on the institutional routine breast cancer imaging protocol. These included a non-contrast-enhanced scan, followed by arterial and equilibrium phases after contrast injection. The contrast medium (300 mgI/mL) was injected at a dose of 2 mL per kilogram body weight, administered over 30 s. The arterial phase was acquired 50 s after injection.

### MRI acquisition

MRI aimed to conduct a preoperative evaluation of tumor extent. MRI images were obtained either at our institution (n = 14) or at external institutions (n = 8). Both 1.5T (n = 1) and 3T (n = 21) scanners from various vendors were included in this analysis.

For the in-house MRI cohort, breast MRI was performed on a 3.0-T system (SIGNA Pioneer, GE HealthCare, Milwaukee, WI, USA) using a dedicated 16-channel breast coil. Dynamic contrast-enhanced imaging was acquired using a 3D T1-weighted fat-suppressed gradient-echo sequence (VIBRANT; TR/TE 5.4/2.6 ms; flip angle 10°; in-plane resolution 0.8 × 1.0 mm; slice thickness 1.0 mm; FOV 360 × 360 mm; axial plane). Gadobutrol (1.0 mmol/mL) was administered at 0.1 mL/kg (0.1 mmol/kg) at 1 mL/s, followed by a 30-mL saline flush. Dynamic phases were obtained pre-contrast and approximately 150 s and 360 s after injection; the 150-s phase was used for DCE-T1WI analysis.

The dynamic contrast-enhanced MRI protocols at the external institutions varied and were performed according to each institution’s routine clinical practice. For consistency, the phase with the best lesion visibility (typically corresponding to the early phase) was retrospectively selected for analysis.

### Objective image analysis

Quantitative analysis of CT scan images was independently performed by two radiologists (4 and 13 years of experience). Circular regions-of-interest (ROIs, 1 cm^2^ when feasible) were placed in the breast lesion, background parenchyma, and ipsilateral pectoralis major muscle; for lesions in which a 1 cm^2^ ROI could not be accommodated, we used the largest circular ROI that fit entirely within the enhancing portion of the lesion on the same slice. Each ROI was measured three times per series, and the mean value was used. Noise was defined as the standard deviation of attenuation in the pectoralis muscle on the same slice. The CNR was calculated as the difference in CT attenuation between the lesion and background breast parenchyma within the ROIs, divided by the noise.

### Image review and subjective analysis

All images were independently reviewed by three readers: Reader 1, a first-year radiology resident; Reader 2, a board-certified radiologist with 8 years of experience in breast and thoracic imaging; and Reader 3, a board-certified radiologist with 11 years of experience in breast and thoracic imaging. For each participant, 40-keV VMI, 70-keV VMI, and DCE-T1WI were evaluated. The initial display window for CT scan was set at a width of 300 HU and a level of 40 HU, which could be freely adjusted by the readers. The readers were aware of the cancer diagnosis but were blinded to the VMI energy level. Image review was conducted across three separate sessions. One session was dedicated to MRI evaluation (DCE-T1WI), and two sessions were used for CT evaluation, where 40-keV and 70-keV VMI datasets were presented in a randomized order. The case order within each session was randomized per reader to reduce ordering effects. To decrease recall bias, reading sessions were separated by at least 2 weeks.

Lesion conspicuity was evaluated as the primary endpoint using a 5-point Likert scale. If the predefined index lesion was not detected on a given modality, it was assigned a conspicuity score of 1 by definition. The secondary and exploratory endpoints included lesion size error, detection rate, subjective image noise, BPE, lesion CNR (from the objective analysis), and reading time. The detection rate (per-lesion sensitivity) was evaluated within a known-positive cohort and was defined as the correct localization of the index lesion. For lesions that were accurately identified, the maximum lesion diameter was determined on axial images (i.e., the largest diameter across axial slices) and absolute size error was calculated as the absolute difference between the maximum imaging diameter (D_imag_) and the maximum pathological diameter (D_patho_), normalized by the pathological size (D_patho_), as follows:

$${\text{Absolute size error}}=\frac{\left|{D}_{imag}-{D}_{patho}\right|}{{D}_{patho}}$$

In addition, a directional size error was calculated to assess the direction of bias, defined as:

$${\text{Directional size error}}=\frac{{D}_{imag}-{D}_{patho}}{{D}_{patho}}$$

Subjective image noise was rated on a 5-point Likert scale. BPE was evaluated on a 3-point scale. This 3-point scale was adapted from the standard 5-point MRI scale to better suit the assessment of CT enhancement patterns. Finally, reading time was measured in seconds, and it was defined as the interval from opening the dataset to the completion of the evaluation.

### Reference standard and lesion correlation

Surgical pathology was the reference standard for all findings. The index lesion for analysis was defined as the largest surgically confirmed malignant lesion in each breast. This lesion was identified from the surgical pathology report before the image review started. The location of imaging findings was correlated with the pathology report based on location and size information. Further, surgical pathology provided the reference standard for the maximum pathological diameter used in size error calculations.

### Statistical analysis

All statistical analyses were performed in Python (version 3.10). A *p* value of < 0.05 indicated statistically significant differences for all analyses.

Statistical comparisons were categorized into two groups. First, metrics that were methodologically specific to CT scan (i.e., not comparable to MRI), particularly lesion CNR and subjective image noise, were compared only between the 40 and 70-keV VMI datasets using the Wilcoxon signed-rank test. Second, the endpoints evaluated across the three imaging datasets (40-keV VMI, 70-keV VMI, and DCE-T1WI) were analyzed using statistical tests appropriate for each variable type. In the primary analyses, scores were summarized at the lesion level by averaging values across the three readers for each lesion and modality (reader-averaged analysis); for size-related endpoints, reader-averaged values were calculated using only readers who detected the lesion in the corresponding modality. The lesion detection score was calculated as the lesion-level average of reader-level binary detections (0/1) and compared across modalities using the Friedman test. The remaining continuous or ordinal endpoints (conspicuity, size error, BPE, and reading time) were also compared using the Friedman test. If the overall test was significant, pairwise post-hoc comparisons were conducted using the Wilcoxon signed-rank test with Holm adjustment. As a sensitivity analysis, reader-averaged comparisons for key endpoints (conspicuity and size error) were repeated in the in-house MRI subgroup using the same statistical framework as the main analysis. For all post-hoc analyses, the adjusted p-values were reported. Inter-reader agreement was assessed for key subjective and reader-dependent endpoints (lesion conspicuity, lesion detection, noise, and BPE) using Fleiss’ κ, calculated on a per-lesion basis across the three readers. Inter-reader agreement for size measurements was assessed using intraclass correlation coefficients (ICC; two-way random-effects, absolute-agreement, single-measurement model [ICC(A,1)]), calculated separately for each modality.

## Results

### Characteristics of the participants

**Fig. 2:**
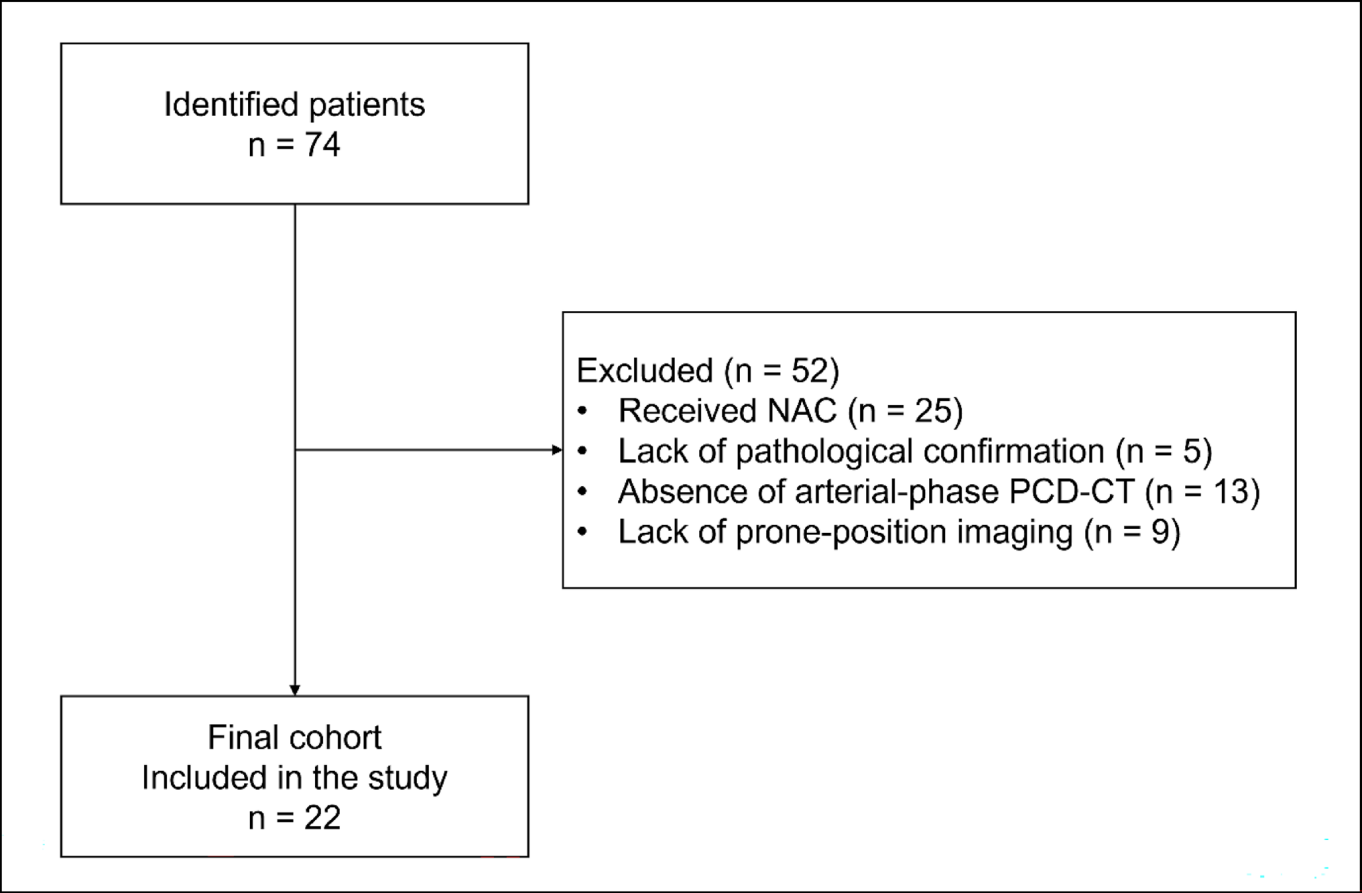
Flowchart showing participant enrollment. *NAC* neoadjuvant chemotherapy, *PCD-CT* photon-counting detector computed tomography

Of the 74 identified participants, 25 were excluded due to NAC, 5 due to lack of pathological confirmation of primary breast cancer, 13 due to the absence of an arterial-phase series in PCD-CT, and 9 due to the lack of either PCD-CT or MRI performed in the prone position. After applying these criteria, 22 women were finally included in the study cohort (median age, 55.5 years [Interquartile Range (IQR) 47.2–63.0]; range 39–84 years). The participant selection process is summarized in Fig. 2. Detailed clinicopathologic characteristics are summarized in Table 1. The median absolute interval between CT and MRI was 14 days (IQR 3–25.5). The median interval from CT to surgery was 52 days (IQR 37–63.75), and that from MRI to surgery was 52 days (IQR 40.5–66). The median signed interval between CT and MRI (CT date minus MRI date) was 1.5 days (IQR − 4.5 to 23.25). CT was performed before MRI in 10/22 cases (45.5%) and after MRI in 12/22 cases (54.5%).

**Table 1:** Characteristics of the patients

Characteristics of the patients	Value
Age (years)	55.5 [47.2–63.0] (range 39–84)
Maximum tumor diameter (mm)	19.0 [15.0–40.0] (range 5.0–85.0); n = 21
Maximum invasive component (mm)	12.0 [5.0–18.8] (range 0.0–60.0); n = 22
Histologic subtype	–
Invasive ductal carcinoma	12 (54.5%)
Ductal carcinoma in situ	5 (22.7%)
Invasive lobular carcinoma	3 (13.6%)
Mucinous carcinoma	1 (4.5%)
Solid papillary carcinoma	1 (4.5%)
ER positivity	21 (95.5%)
PR positivity	20 (90.9%)
HER2 positivity	1 (4.5%)
Ki-67 ≥ 20%	6 (27.3%)
Molecular subtype	–
Luminal A-like	15 (68.2%)
Luminal B-like/HER2-negative	5 (22.7%)
Luminal B-like/HER2-positive	1 (4.5%)
Triple-negative	0 (0%)
HER2-enriched	0 (0%)
Unknown	1 (4.5%)

Values were presented as the median [IQR] unless otherwise indicated. Range values were provided in the parentheses

Information on tumor diameter was unavailable for one case; thus, this case was excluded from the corresponding analysis (n = 21)

*ER* estrogen receptor, *PR* progesterone receptor, *HER2* human epidermal growth factor receptor 2, *IQR* interquartile range

### Lesion conspicuity

**Fig. 3:**
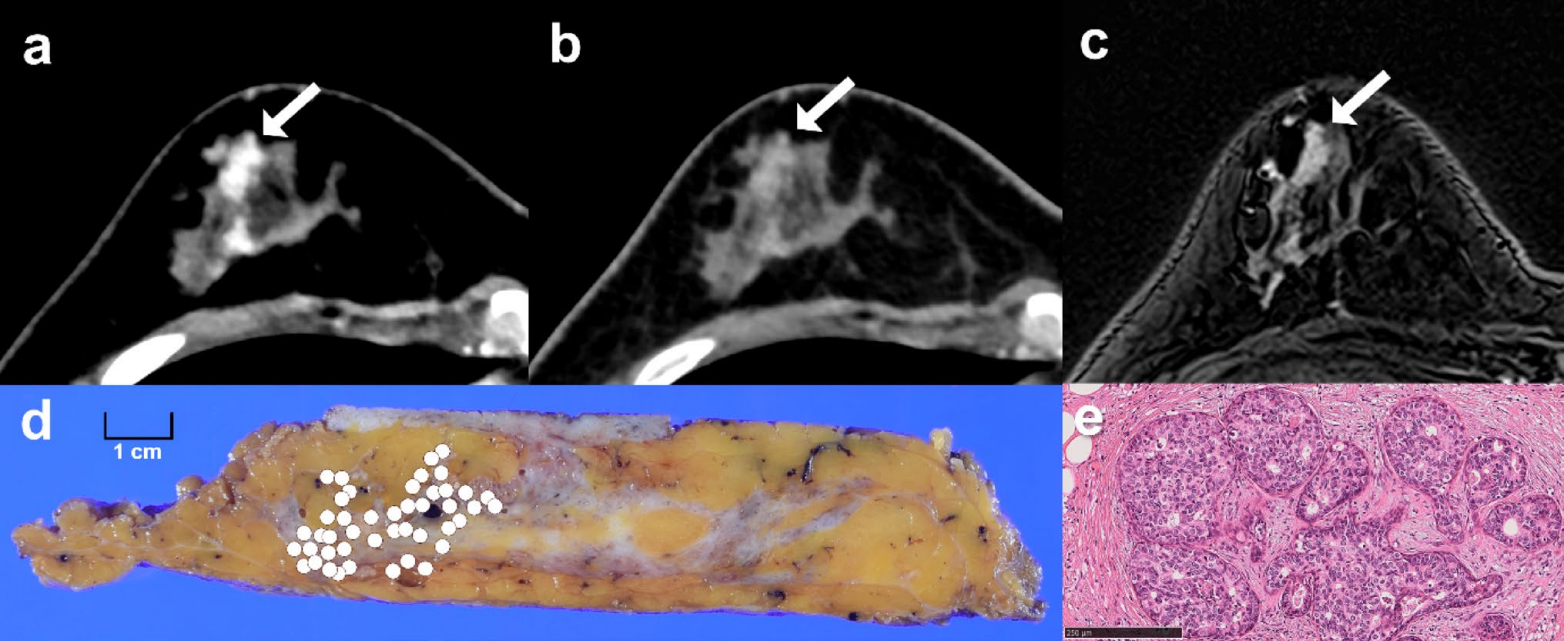
Representative case of ductal carcinoma in situ (DCIS) in a 56-year-old woman. **a** Axial 40-keV virtual monoenergetic image (VMI) obtained in the arterial phase shows an intensely enhancing mass (arrow). **b** Axial 70-keV VMI at the same level. The lesion (arrow) shows lower attenuation and is less conspicuous compared to the 40-keV image. **c** Axial dynamic contrast-enhanced T1-weighted imaging (DCE-T1WI) shows the corresponding enhancing mass (arrow). The mean subjective conspicuity scores for this case were 4.7 for 40-keV VMI, 2.7 for 70-keV VMI, and 2.7 for DCE-T1WI. **d** Photograph of the cut surface of the resected specimen. The extent of the DCIS lesion is mapped with white dots. **e** Photomicrograph of the histopathological specimen (hematoxylin and eosin stain)

**Fig. 4:**
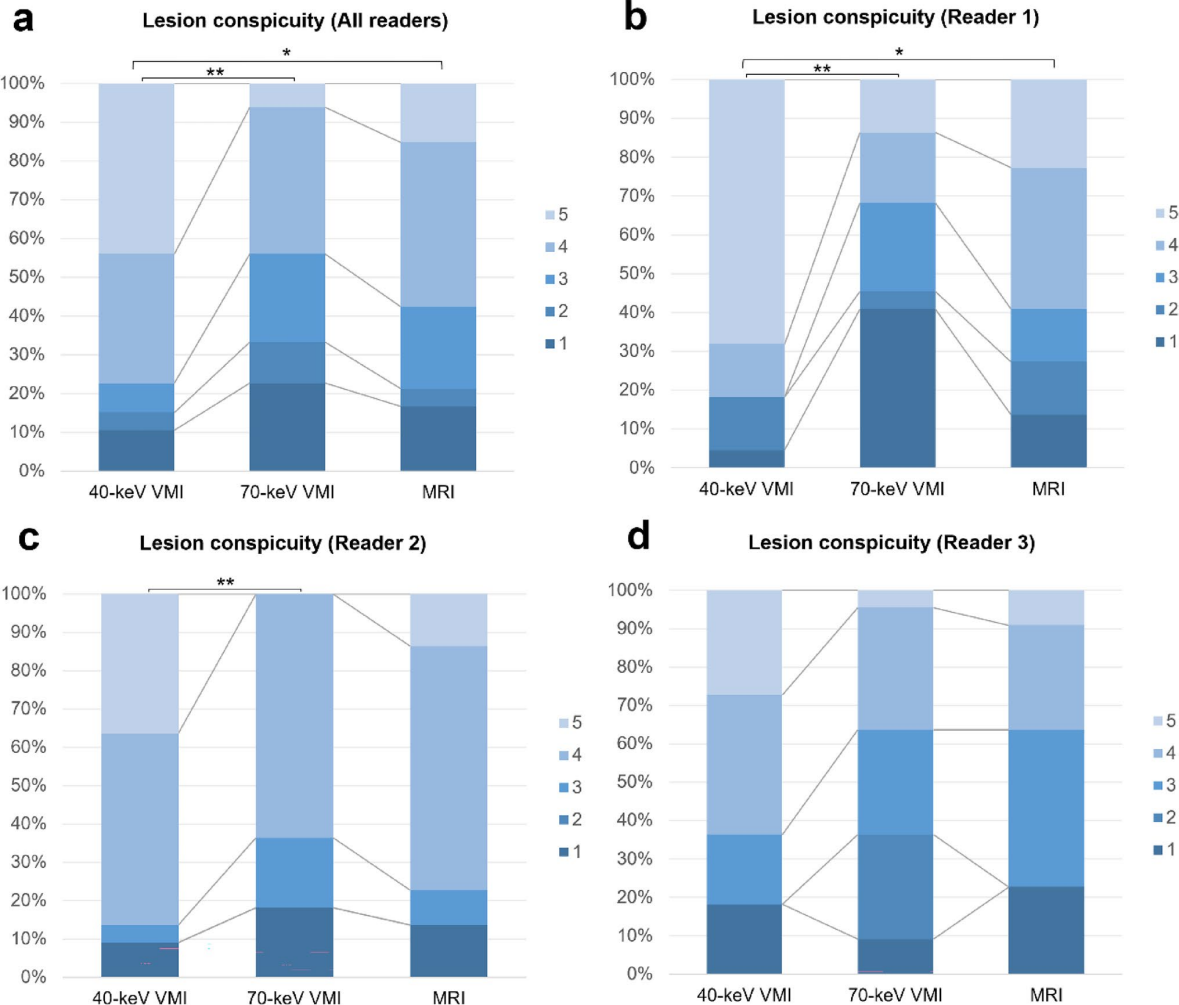
Lesion conspicuity distributions across modalities. Stacked 100% bar charts show the Likert scores (1–5; undetected lesions counted as 1) for 40-keV virtual monoenergetic images (VMIs), 70-keV VMI, and MRI in the pooled cohort (**a**) and by the reader (**b**–**d**). Friedman test, *p* < 0.001; pairwise Wilcoxon signed-rank tests with Holm adjustment: 40-keV vs. 70-keV, adjusted *p* = 0.002; 40-keV vs. MRI, adjusted *p* = 0.041; 70-keV vs. MRI, adjusted *p* = 0.098. Pooled analysis was performed at the lesion level using reader-averaged scores. Statistically significant pairwise differences are marked with asterisks (*adjusted *p* < 0.05; **adjusted *p* < 0.01)

Figure 3 shows a representative case. The lesion conspicuity scores, as assessed on a 5-point scale, differed significantly among the three datasets (*p* < 0.001, the Friedman test). In the lesion-level reader-averaged analysis (n = 22), conspicuity differed significantly among modalities (Friedman test, *p* < 0.001). Median reader-averaged conspicuity scores [IQR] were 4.33 [3.67–4.67] for 40-keV VMI, 2.83 [2.33–3.67] for 70-keV VMI, and 3.67 [2.42–4.00] for MRI. Pairwise comparisons showed higher conspicuity for 40-keV than for 70-keV (*p* = 0.002) and MRI (*p* = 0.041), while 70-keV vs. MRI was not significant (*p* = 0.098). (Fig. 4a).

Reader 1 showed significant differences among modalities (*p* < 0.001), with higher scores for 40-keV VMI relative to 70-keV VMI (*p* = 0.001) and DCE-T1WI (*p* = 0.030) (Fig. 4b). For Reader 2, the overall variations among modalities were significant (*p* = 0.007), and 40-keV VMI had a higher conspicuity than 70-keV VMI (*p* = 0.008). However, no significant differences were observed in terms of conspicuity between 40-keV VMI and DCE-T1WI (*p* = 0.368) or between 70-keV VMI and MRI (*p* = 0.368) (Fig. 4c). Reader 3 also demonstrated significant overall differences (*p* = 0.015). However, the pairwise comparison did not reach statistical significance (all *p* > 0.05) (Fig. 4d).

Inter-reader agreement for lesion conspicuity, assessed using Fleiss’ κ, was fair overall (κ = 0.24 across all datasets), with comparable values for 40-keV VMI (κ = 0.28) and lower agreement for 70-keV VMI (κ = 0.10).

To assess the robustness of the primary conspicuity findings to reader-dependent scoring behavior, we performed a leave-one-reader-out sensitivity analysis (Online Resource 1). Across all three leave-one-reader-out analyses (excluding Reader 1, Reader 2, or Reader 3), the overall modality difference remained significant (Friedman *p* < 0.001), and 40-keV VMI consistently showed higher conspicuity than 70-keV VMI (*p* < 0.013). In contrast, the 40-keV VMI vs. DCE-T1WI comparison was sensitive to reader exclusion, with Holm-adjusted *p* values ranging from 0.012 to 0.068.

### Lesion size error

The lesion size error was analyzed only for lesions that were accurately identified and for which the maximum pathological diameter was available, enabling valid comparisons across all modalities. One participant did not undergo measurement of pathological tumor size (Table 2).

**Table 2:** Lesion size error by imaging dataset

Imaging dataset	Absolute size error	Directional size error
	Median [IQR] (%)*	Median [IQR] (%)*
40-keV VMI	35.7 [20.0–60.8]	− 29.2 [− 54.4 to − 15.8]
70-keV VMI	22.5 [14.1–55.6]	− 22.1 [− 55.6 to − 6.7]
DCE-T1WI	18.0 [13.3–61.0]	− 15.4 [− 52.1 to − 2.7]

Pairwise comparison	Δ median [IQR] (%)	*p* value	Δ median [IQR] (%)	*p* value
Overall difference		0.230**		0.045**
40-keV vs. 70-keV	–	–	− 2.1 [− 7.7 to − 1.3]	0.222***
40-keV vs. DCE-T1WI	–	–	− 12.2 [− 16.0 to − 0.2]	0.009***
70-keV vs. DCE-T1WI	–	–	− 4.2 [− 11.4 to − 2.4]	0.075***

*keV* kiloelectron volt, *VMI* virtual monoenergetic image, *DCE-T1WI* dynamic contrast-enhanced T1-weighted imaging

*Pathological and imaging maximum diameters were measured in mm; size errors are reported as normalized percentages. Size error was defined as (imaging maximum diameter − pathological maximum diameter)/pathological maximum diameter. Absolute size error was the absolute value of size error, whereas directional size error retained the sign (negative values indicate underestimation). Lesion-level values were averaged across only the readers who detected the lesion in the corresponding modality. Cross-modality comparisons were restricted to lesions with pathological size available and evaluable lesion-level values in all three modalities (n = 18)

**Friedman test. Post hoc pairwise tests were not performed because the overall test was not significant

***Wilcoxon signed-rank test

For cross-modality comparisons, analyses were restricted to lesions that had size measurements available in all three modalities, defined as being detected by at least one reader in each modality (complete-case set; n = 18 lesions). Using lesion-level averages, median absolute normalized size errors [IQR] were 35.7% [20.0–60.8] for 40-keV VMI, 22.5% [14.1–55.6] for 70-keV VMI, and 18.0% [13.3–61.0] for DCE-T1WI. The overall modality difference was not significant (Friedman test, *p* = 0.230).

Directional size error analysis, based on the same strategy, showed negative medians across modalities, indicating a tendency toward underestimation: − 29.2% [IQR − 54.4 to − 15.8] for 40-keV VMI, − 22.1% [− 55.6 to − 6.7] for 70-keV VMI, and − 15.4% [− 52.1 to − 2.7] for DCE-T1WI. The overall modality effect was significant (Friedman *p* = 0.045); in pairwise testing, 40-keV vs. DCE-T1WI was significant (*p* = 0.009), whereas 40-keV vs. 70-keV (*p* = 0.222) and 70-keV vs. DCE-T1WI (*p* = 0.075) were not.

Inter-reader agreement for lesion long-axis size measurements varied by modality. ICC(A,1) values were 0.70 for 40-keV VMI (n = 14 lesions), 0.94 for 70-keV VMI (n = 7), and 0.88 for DCE-T1WI (n = 13).

### Detection rate

In lesion-level reader-averaged analyses (n = 22), overall detection proportions differed across modalities (Friedman test, *p* = 0.025). Mean detection proportions were 90.9% for 40-keV VMI, 77.3% for 70-keV VMI, and 84.8% for DCE-T1WI (Table 3). However, pairwise differences were not statistically significant (40-keV vs. 70-keV, *p* = 0.061; 40-keV vs. DCE-T1WI, *p* = 0.503; 70-keV vs. DCE-T1WI, *p* = 0.503). Inter-reader agreement for lesion detection was moderate overall (κ = 0.43) and was higher at 40-keV VMI (κ = 0.63) than at 70-keV VMI (κ = 0.31).

**Table 3:** Detection rate across imaging datasets

Imaging dataset	Detection rate (%)*
40-keV VMI	90.9 (60/66)
70-keV VMI	77.3 (51/66)
DCE-T1WI	84.8 (56/66)

Pairwise comparison	*p* value
Overall difference	0.025**
40-keV vs. 70-keV	0.061***
40-keV vs. DCE-T1WI	0.503***
70-keV vs. DCE-T1WI	0.503***

*keV* kiloelectron volt, *VMI* virtual monoenergetic image, *DCE-T1WI* dynamic contrast-enhanced T1-weighted imaging

*Detection score was defined as correct localization of the index lesion within a known-positive cohort and was summarized at the lesion level by averaging binary reader detections (0/1) across three readers; per modality, n = 22 lesions

**Friedman test

***Wilcoxon signed-rank tests (Holm-adjusted)

### Subjective image quality

**Fig. 5:**
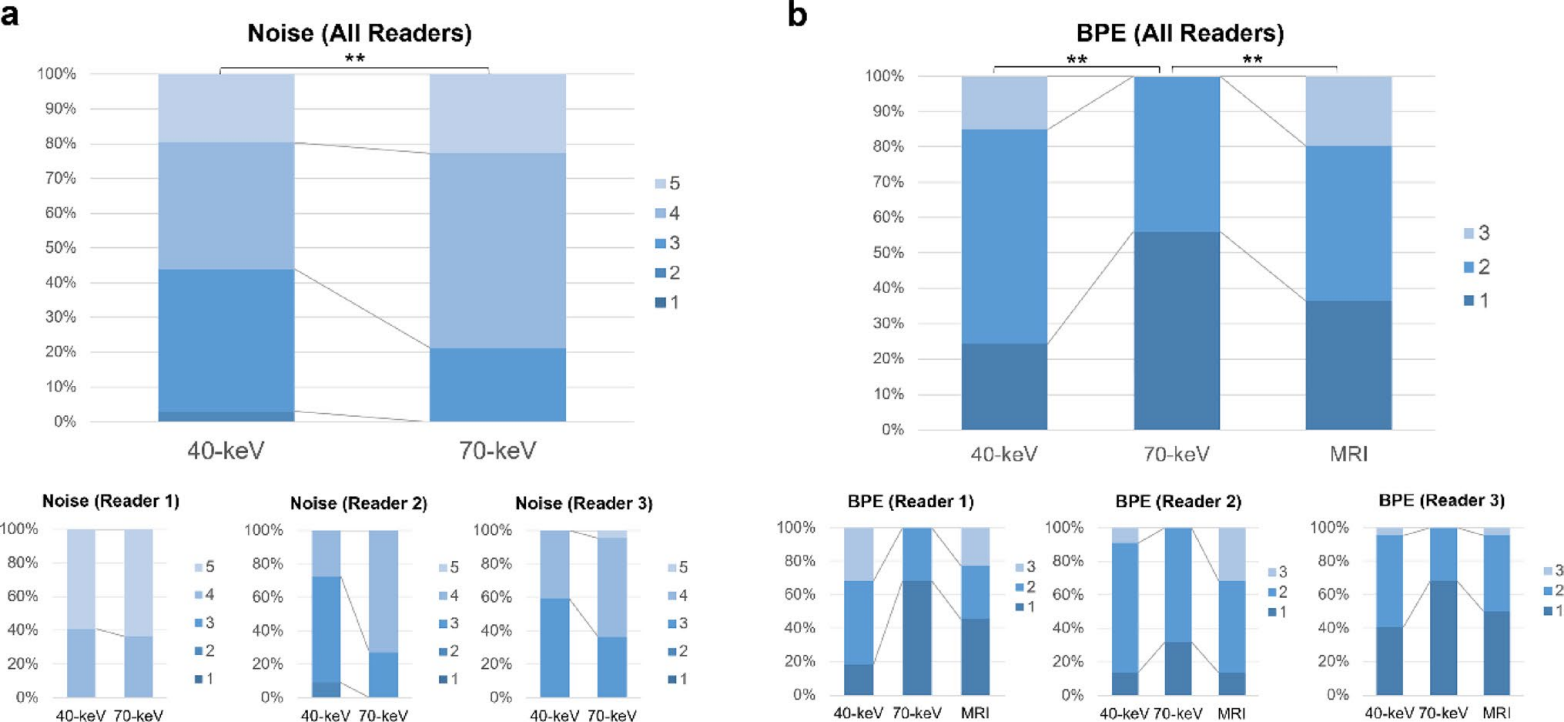
Subjective image quality. **a** Noise displayed as 100% stacked bar charts of the 5-point Likert scores (1 = severe, 5 = none) for 40-keV and 70-keV virtual monoenergetic images (VMIs) (22 lesions × 3 readers = 66 observations per modality); lower panels show reader-specific distributions. For inferential comparison, lesion-level reader-averaged scores were used (n = 22 lesions). The scores were lower for 40-keV than for 70-keV, indicating greater perceived noise at 40-keV (median [IQR] 3.67 [3.67–4.00] vs. 4.17 [3.67–4.33]; Wilcoxon signed-rank test, *p* = 0.009). **b** Background parenchymal enhancement (BPE) shown as 100% stacked bar charts of the 3-point Likert scores (1 = none or minimal, 2 = mild to moderate, and 3 = marked) for 40-keV VMI, 70-keV VMI, and MRI (22 lesions × 3 readers = 66 observations per modality); lower panels show reader-specific distributions. Pairwise comparisons: 40-keV > 70-keV (*p* = 0.003), MRI > 70-keV (*p* = 0.008), and 40-keV vs. MRI not significant (*p* = 0.384). Statistically significant pairwise differences are marked with asterisks (*p < 0.05; **p < 0.01)

Subjective noise was assessed on a 5-point scale (1, severe; 5, none). In lesion-level reader-averaged analyses, subjective noise (40-keV vs. 70-keV) differed significantly (Wilcoxon signed-rank test, *p* = 0.009). Median reader-averaged noise scores [IQR] were 3.67 [3.67–4.00] for 40-keV VMI and 4.17 [3.67–4.33] for 70-keV VMI, indicating greater perceived noise at 40-keV (Fig. 5a).

BPE was assessed on a 3-point scale (1, none or minimal; 2, mild to moderate; and 3, marked). For BPE, significant overall differences were observed across modalities (Friedman test, *p* < 0.001). Median reader-averaged BPE scores [IQR] were 2.00 [1.67–2.00] for 40-keV VMI, 1.50 [1.33–1.67] for 70-keV VMI, and 1.67 [1.42–2.25] for DCE-T1WI. Pairwise comparisons showed higher BPE for 40-keV vs. 70-keV (*p* = 0.003) and DCE-T1WI vs. 70-keV (*p* = 0.008), while 40-keV vs. DCE-T1WI was not significant (*p* = 0.384) (Fig. 5b).

Inter-reader agreement for subjective image quality metrics was limited (poor for noise, κ = − 0.10; slight for BPE, κ = 0.11). Given the low inter-reader agreement, reader-specific score distributions for subjective noise and BPE are also provided in Fig. 5.

### Lesion CNR

**Fig. 6:**
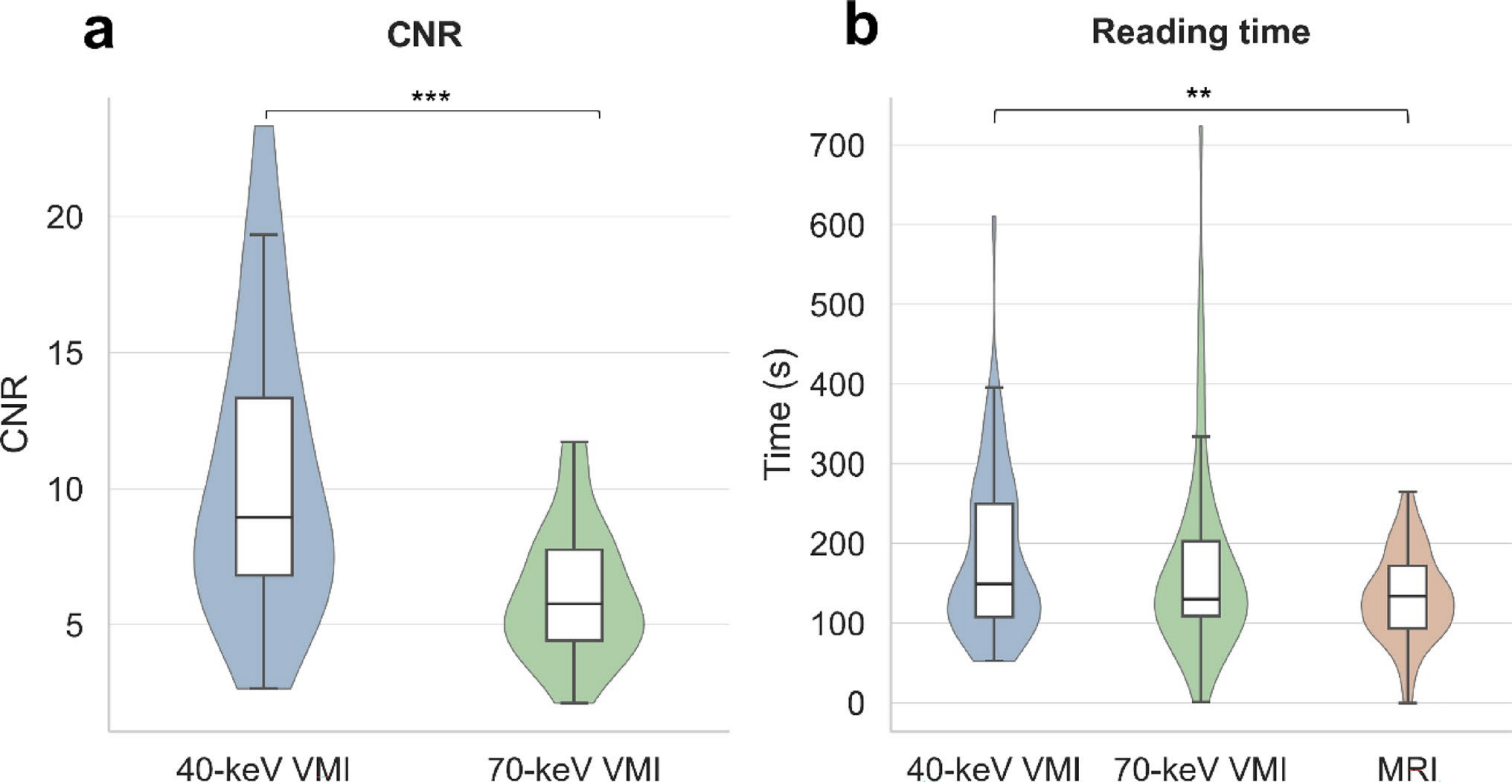
Quantitative metrics: lesion CNR and reading time. **a** Lesion CNR for virtual monoenergetic images (VMIs). The CNR was higher at 40-keV VMI than at 70-keV VMI (median 8.95 [IQR 6.81–13.34] vs. 5.78 [4.41–7.75]; Wilcoxon signed-rank test, *p* < 0.001; Δmedian = 4.4 [IQR 2.1–6.2]). **b** Reading time across modalities. The overall differences were significant (Friedman test, *p* = 0.001); 40-keV VMI required a longer reading time than MRI (*p* = 0.001), whereas 40-keV vs. 70-keV VMI (*p* = 0.284) and 70-keV VMI vs. MRI (*p* = 0.238) were not significant. Statistically significant pairwise differences are marked with asterisks (**p < 0.01; ***p < 0.001)

Lesion CNR, evaluated only between the VMI datasets, was significantly higher at 40-keV than at 70-keV (median, 8.95 [IQR 6.81–13.34] vs. 5.78 [4.41–7.75]; Wilcoxon signed-rank test, *p* < 0.001). The difference in terms of medians was 4.4 (IQR 2.1–6.2) (Fig. 6a).

### Reading time

The reading time differed significantly among modalities in lesion-level reader-averaged analysis (Friedman test, *p* = 0.001). Median reader-averaged reading times [IQR] were 174.3 [157.2–203.8] s for 40-keV VMI, 131.0 [119.1–186.3] s for 70-keV VMI, and 128.8 [118.3–155.7] s for DCE-T1WI. In pairwise comparisons, 40-keV VMI required longer reading time than DCE-T1WI (adjusted *p* = 0.001), while 40-keV vs. 70-keV (adjusted *p* = 0.284) and 70-keV vs. DCE-T1WI (adjusted *p* = 0.238) were not significant (Fig. 6).

### Subgroup analysis

To assess robustness to MRI heterogeneity, we repeated reader-averaged analyses in the in-house MRI subgroup (n = 15). The overall conspicuity difference remained significant (Friedman *p* = 0.001), and 40-keV VMI remained superior to 70-keV VMI (*p* = 0.011); however, the 40-keV vs. MRI difference was not significant in this subgroup (*p* = 0.218), unlike in the overall cohort (*p* = 0.041).

For size error, absolute normalized size error did not differ across modalities in either the overall cohort (*p* = 0.230; n = 18) or the in-house subgroup (*p* = 0.320; n = 13). In directional size error, the 40-keV vs. MRI pairwise difference was significant in the overall cohort (*p* = 0.009) but not in the in-house subgroup (*p* = 0.062).

## Discussion

The current study suggests that 40-keV VMI may be a valuable tool when lesion conspicuity and reliable localization are the primary clinical objectives. However, this was accompanied by a modest increase in reading time. In addition, directional size error analysis suggested a tendency toward underestimation, with 40-keV VMI showing greater underestimation than DCE-T1WI. Because acquisition timing differed between arterial-phase PCD-CT and DCE-T1WI, this phase mismatch may have contributed to differences in size measurements.

The findings of this study are in accordance with those of previous spectral CT studies on breast cancer, which consistently showed that low-keV virtual monoenergetic images, particularly at approximately 40-keV, yielded superior tumor conspicuity and improved lesion detectability compared with conventional CT scan images [6, 7, 21, 22]. The observed benefit of low-energy VMI is consistent with reports in the spectral CT literature, which show that energies near 40-keV enhance iodine-related contrast and improve conspicuity [23–26]. The mechanistic basis reflects the closeness to the iodine K-edge at 33.2-keV, which maximizes lesion-to-background signal [5]. A key feature of this study is performing a comparison in the same participant on a PCD-CT system against DCE-MRI. In particular, it focused on arterial-phase PCD-CT that reflects tumor vascularity and early enhancement dynamics. The superior energy discrimination and the fine spatial sampling of the PCD-CT system likely helped preserve low-energy contrast while limiting electronic noise compared with traditional detectors that integrate energy, which is consistent with the higher conspicuity and CNR measured at 40-keV.

According to these findings, a clinical application based on the specific objective can be proposed. Previous studies have highlighted limitations of MRI in the assessment of minute lesions, underscoring the need for a multimodality imaging approach [27]. In this context, the 40-keV VMI protocol may be particularly useful when the priority is maximizing conspicuity for localization. The typical scenarios include participants who cannot undergo MRI, time-sensitive preoperative workups when MRI access is limited, CT scan-based staging pathways where a breast protocol can be added, and cases with substantial MRI artifacts from devices or clips [28]. However, MRI remains the reference standard when precise size estimation and assessment of complex disease extent are the primary goals, as arterial-phase CT may underestimate lesion extent.

The current study had several limitations that should be acknowledged. First, this was a single-center study with a small sample size (n = 22), and this could have limited statistical power. In addition, prone-position PCD-CT was selectively performed in clinical practice for participants requiring detailed local assessment; therefore, the study cohort represents a clinically selected population rather than an unselected staging cohort. As a result, the generalizability of the findings is inherently limited, and larger, more diverse cohorts are required to validate these results. Nevertheless, this cohort reflects a real-world clinical scenario in which prone PCD-CT is requested for detailed local assessment, and the present findings may be most applicable to such participants. Additionally, the distribution of histologic subtypes (e.g., invasive lobular carcinoma or mucinous carcinoma) may have influenced lesion conspicuity, detection, and size estimation; this should be examined in larger cohorts.

Second, the inclusion of both in-house and external MRI examinations introduced significant protocol differences (including field strength, temporal resolution, and sequence parameters). These examinations were performed according to each institution’s routine clinical protocols; therefore, the MRI comparator in this study represents a real-world rather than a standardized, optimized breast MRI setting. In a sensitivity analysis restricted to in-house MRI cases (n = 15), the pairwise difference in lesion conspicuity between 40-keV VMI and MRI was not statistically significant. This variability might not reflect the full capabilities of a dedicated protocol and could have biased the comparison in favor of PCD-CT. Further, our comparison evaluated single arterial-phase PCD-CT against the peak enhancement phase of DCE-MRI, rather than strictly matching the temporal phase, and this phase selection may also have introduced bias. The in-house DCE-MRI images were obtained at approximately 150 s after contrast injection, slightly later than the typical early phase (60–120 s), which may have affected lesion conspicuity and BPE on MRI and thus the cross-modality comparison. Thus, our findings do not indicate that single-phase 40-keV VMI can replace the comprehensive information from multiphase DCE-MRI, particularly for assessing non-mass enhancement or detailed enhancement patterns over time.

Inter-reader agreement varied across endpoints and represents a study limitation. Agreement was moderate for size measurements and lesion detection but lower for subjective assessments such as conspicuity, BPE, and image noise. In a leave-one-reader-out sensitivity analysis, the statistical significance of the 40-keV VMI vs. T1WI conspicuity comparison varied depending on which reader was excluded (Online Resource 1). Therefore, this comparison should be interpreted with caution, particularly given the limited inter-reader agreement for subjective scoring. The small subset of lesions available for size-error analysis was another limitation. This subset was limited because lesions must be correctly identified on all modalities and have pathological data, which reduced the precision of these estimates. Finally, our measurements reflected per-lesion sensitivity in a known-positive cohort. Hence, specificity and overall diagnostic accuracy were not assessed.

In the future, prospective multicenter studies with standardized MRI protocols and larger cohorts should be performed. Further technical optimization of PCD-CT for low-keV imaging, including reconstruction methods and denoising tailored to 40-keV, may help mitigate noise and shorten reading time [29–32]. The evaluations of multiphase PCD-CT or methods that combine low- and high-keV information could validate optimal roles for detection versus measurement and allow a more direct alignment with the full dynamic enhancement data from DCE-MRI. Finally, beyond low-keV VMI-based conspicuity, PCD-CT may offer additional value through improved depiction of calcifications [15] and quantitative iodine mapping [17, 18]. These capabilities were not evaluated in the current study and warrant dedicated validation in future prospective studies.

In conclusion, arterial-phase 40-keV VMI from prone PCD-CT showed the highest lesion conspicuity. Its overall performance was favorable relative to DCE-T1WI, although pairwise differences were not consistently statistically significant. These findings support a complementary role for 40-keV VMI in preoperative breast assessment and highlight its potential within PCD-CT–based staging pathways to deliver both systemic evaluation and high-quality local breast assessment in a single session. MRI remains the reference standard for comprehensive local evaluation, and arterial-phase CT may underestimate lesion extent.

## Supplementary Information

Below is the link to the electronic supplementary material.

Supplementary Material 1
