# Supplementary material for: Lesion conspicuity assessment of prone 40-keV virtual monoenergetic imaging in arterial-phase photon-counting CT for breast cancer: a retrospective comparison with DCE-MRI
Source: Jpn J Radiol. 2026 Mar 31;44(7):1177–88. doi: 10.1007/s11604-026-01979-7 (PMC13315485; doi:10.1007/s11604-026-01979-7)
Supplement: Supplementary file 1 — Supplementary Material 1 [file 11604_2026_1979_MOESM1_ESM.pdf]

Supplementary Material (Online Resource 1) — Japanese Journal of Radiology
Article: Lesion Conspicuity Assessment of Prone 40-keV Virtual Monoenergetic Imaging in Arterial-phase Photon-counting CT for Breast Cancer: A Retrospective Comparison with DCE-MRI
Authors: Yusuke Kawasaki, Maika Nakajima, Kota Yokoyama, Emi Yamaga, Makoto Kato, Leona Katsuta, Ken Yamagiwa, Hiroto Hada, Takumi Hiraishi, Hirofumi Yamada, Junichi Tsuchiya, Susumu Kirimura, Iichiroh Onishi, Tomoyuki Aruga, Ukihide Tateishi
Corresponding author: Kota Yokoyama (kota1986ky@yahoo.co.jp)

Supplementary Table S1. Leave-one-reader-out sensitivity analysis for lesion conspicuity

	Conspicuity score*			Overall difference	Pairwise comparison		
Analysis set	40-keV VMI Median [IQR]	70-keV VMI Median [IQR]	DCE-T1WI Median [IQR]	p value (Friedman test)	40-keV vs 70-keV p value	40-keV vs DCE-T1WI p value	70-keV vs DCE-T1WI p value
All 3 readers (primary)	4.33 [3.67–4.67]	2.83 [2.33–3.67]	3.67 [2.42–4.00]	0.0001	0.0020	0.0414	0.0982
Excluding Reader 1	4.00 [3.50–4.50]	3.25 [2.62–4.00]	3.50 [3.00–4.00]	0.0008	0.0123	0.0678	0.2313
Excluding Reader 2	4.50 [3.00–4.88]	2.50 [2.00–3.50]	3.50 [2.50–4.00]	0.0002	0.0048	0.0125	0.1326
Excluding Reader 3	4.50 [4.00–5.00]	3.25 [2.00–3.88]	4.00 [3.00–4.00]	0.0001	0.0003	0.0556	0.0624

*Lesion conspicuity was assessed on a 5-point Likert scale (1–5; undetected lesions counted as 1). Analyses were performed at the lesion level using reader-averaged scores. The lesion set was identical across leave-one-reader-out analyses (n = 22). Overall differences among modalities were assessed using the Friedman test, followed by pairwise Wilcoxon signed-rank tests with Holm adjustment.
Abbreviations: keV = kiloelectron volt; VMI = virtual monoenergetic image; DCE-T1WI = dynamic contrast-enhanced T1-weighted imaging.
